# Genome wide association study of HTLV-1–associated myelopathy/tropical spastic paraparesis in the Japanese population

**DOI:** 10.1073/pnas.2004199118

**Published:** 2021-03-01

**Authors:** Marina Penova, Shuji Kawaguchi, Jun-ichirou Yasunaga, Takahisa Kawaguchi, Tomoo Sato, Meiko Takahashi, Masakazu Shimizu, Mineki Saito, Kunihiro Tsukasaki, Masanori Nakagawa, Norihiro Takenouchi, Hideo Hara, Eiji Matsuura, Satoshi Nozuma, Hiroshi Takashima, Shuji Izumo, Toshiki Watanabe, Kaoru Uchimaru, Masako Iwanaga, Atae Utsunomiya, Yasuharu Tabara, Richard Paul, Yoshihisa Yamano, Masao Matsuoka, Fumihiko Matsuda

**Affiliations:** ^a^Center for Genomic Medicine, Kyoto University Graduate School of Medicine, Sakyo-ku, 606-8507 Kyoto, Japan;; ^b^Laboratory of Virus Control, Institute for Virus Research, Kyoto University, Sakyo-ku, 606-8507 Kyoto, Japan;; ^c^Department of Rare Diseases Research, Institute of Medical Science, St. Marianna University School of Medicine, Miyamae-ku, Kawasaki, 216-8511 Kanagawa, Japan;; ^d^Department of Microbiology, Kawasaki Medical School, Kurashiki, 701-0192 Okayama, Japan;; ^e^Department of Hematology, International Medical Center, Saitama Medical University, Hidaka, 350-1298 Saitama, Japan;; ^f^Department of Neurology, Kyoto Prefectural University of Medicine Graduate School of Medical Science, Kamigyo-ku, 602-8566 Kyoto, Japan;; ^g^Department of Microbiology, Kansai Medical University, Hirakata, 573-1010 Osaka, Japan;; ^h^Division of Neurology, Department of Internal Medicine, Saga University Faculty of Medicine, Saga, 849-8501 Saga, Japan;; ^i^Department of Neurology and Geriatrics, Kagoshima University Graduate School of Medical and Dental Sciences, Kagoshima, 890-8544 Kagoshima, Japan;; ^j^Department of Molecular Pathology, Center for Chronic Viral Diseases, Kagoshima University, Kagoshima, 890-8544 Kagoshima, Japan;; ^k^The Institute of Medical Science and Future Center Initiative, The University of Tokyo, Kashiwa, 277-0871 Chiba, Japan;; ^l^Department of Computational Biology and Medical Sciences, Graduate School of Frontier Sciences, The University of Tokyo, Minato-ku, 108-8639 Tokyo, Japan;; ^m^Department of Frontier Life Sciences, Unit of Basic Medical Sciences, Nagasaki University Graduate School of Biomedical Sciences, Nagasaki, 852-8588 Nagasaki, Japan;; ^n^Department of Hematology, Imamura General Hospital, Kagoshima, 890-0064 Kagoshima, Japan;; ^o^Institut Pasteur–Kyoto University International Mixed Research Unit for Vaccinomics, Institut Pasteur, 75015 Paris, France;; ^p^Department of Hematology, Rheumatology, and Infectious Diseases, Graduate School of Medical Sciences, Faculty of Life Sciences, Kumamoto University, Chuo-ku, 860-8556 Kumamoto, Japan

**Keywords:** HTLV-1, HAM/TSP, HLA, proviral load, genome-wide association study

## Abstract

HTLV-1–associated myelopathy (HAM/TSP) is a chronic and progressive inflammatory disease of the central nervous system. The aim of our study was to identify genetic determinants related to the onset of HAM/TSP in the Japanese population. We conducted a genome-wide association study comprising 753 HAM/TSP patients and 899 asymptomatic HTLV-1 carriers. We also performed comprehensive genotyping of *HLA-A*, *-B*, *-C*, *-DPB1*, *-DQB1*, and *-DRB1* genes using next-generation sequencing technology for 651 HAM/TSP patients and 804 carriers. A strong association was observed in *HLA* class I (*P* = 1.54 × 10^−9^) and class II (*P* = 1.21 × 10^−8^) loci with HAM/TSP. Association analysis using *HLA* genotyping results showed that HLA-C*07:02 (*P* = 2.61 × 10^−5^), HLA-B*07:02 (*P* = 4.97 × 10^−10^), HLA-DRB1*01:01 (*P* = 1.15 × 10^−9^) and HLA-DQB1*05:01 (*P* = 2.30 × 10^−9^) were associated with disease risk, while HLA-B*40:06 (*P* = 3.03 × 10^−5^), HLA-DRB1*15:01 (*P* = 1.06 × 10^−5^) and HLA-DQB1*06:02 (*P* = 1.78 × 10^−6^) worked protectively. Logistic regression analysis identified amino acid position 7 in the G-BETA domain of HLA-DRB1 as strongly associated with HAM/TSP (*P* = 9.52 × 10^−10^); individuals homozygous for leucine had an associated increased risk of HAM/TSP (odds ratio, 9.57), and proline was protective (odds ratio, 0.65). Both associations were independent of the known risk associated with proviral load. DRB1-GB-7-Leu was not significantly associated with proviral load. We have identified DRB1-GB-7-Leu as a genetic risk factor for HAM/TSP development independent of proviral load. This suggests that the amino acid residue may serve as a specific marker to identify the risk of HAM/TSP even without knowledge of proviral load. In light of its allele frequency worldwide, this biomarker will likely prove useful in HTLV-1 endemic areas across the globe.

## Significance

Human T cell leukemia virus type 1 (HTLV-1) proviral load is associated with the risk of developing HTLV-1–associated myelopathy/tropical spastic paraparesis (HAM/TSP) and several small-scale candidate gene approaches have also identified associations of particular *HLA* alleles with HAM/TSP risk. However, no large-scale genome-wide association (GWA) studies have been performed to date. By a large-scale GWA study and comprehensive genotyping of classical *HLA* genes, we found that *HLA-DRB1* alleles carrying leucine at the antigen presentation groove domain (DRB1-GB-7-Leu) increased the susceptibility to HAM/TSP. Individuals who were homozygous for DRB1-GB-7-Leu had a ninefold increased odds of developing HAM/TSP. This effect of DRB1-GB-7-Leu was independent of proviral load. These findings identify DRB1-GB-7-Leu as a genetic risk marker of HAM/TSP development.

The human T cell leukemia virus type 1 (HTLV-1), the first discovered human retrovirus (1, 2), causes adult T cell leukemia (3) and HTLV-1–associated myelopathy/tropical spastic paraparesis (HAM/TSP) (4, 5). There are an estimated 5 to 10 million infected people worldwide (6), of whom ∼1.08 million are in Japan (7). HAM/TSP is chronic and slowly progressive meningomyelitis of the white and gray matter of the central nervous system, causing gait disturbance, leg weakness, back pain, bladder/bowel and sexual dysfunction, and, over time, inability to walk (8). The prevalence of HAM/TSP in the HTLV-1–seropositive population differs among ethnicities, for example ∼0.25% in the Japanese population and ∼1.9% in the Caribbean population (9, 10). This suggests the involvement of virus and host genetic background, although the cause of disease onset is unclear. A higher proviral load in peripheral blood leukocytes is considered a risk factor (11).

Previous studies aiming to identify genetic determinants of HAM/TSP have focused on *HLA* genes. HLA-A*24, HLA-B*07, HLA-C*07, HLA-DQB1*05, and HLA-DRB1*01, as well as a haplotype consisting of these alleles, have been reported to be associated with HAM/TSP in the Japanese population (12, 13). Related studies in other populations have also shown associations of HLA-B*07 and HLA-DRB1*01 with HAM/TSP in a Spanish population (14), HLA-DQB1*05 and HLA-DRB1*01 with HAM/TSP in an Iranian population (15), and HLA-C*07 with HAM/TSP in a Brazilian population (16). By contrast, HLA-A*02 and HLA-C*08 were reported to be protective against HAM/TSP in a Japanese population (13). Another study of a southern Japanese population showed that a lower frequency of HLA-B*40:06 in HAM/TSP patients than in HTLV-1–infected asymptomatic carriers (17). HLA-DQB1*06:02 and HLA-DRB1*15:01 were also shown to work protectively in a population of African descent (18). However, those studies all used hypothesis-dependent target gene approaches focusing on the *HLA* genes and involving relatively small numbers of patients (9 to 232 patients for class I typing and 12 to 195 patients for class II typing).

We organized a multicenter consortium to collect DNA samples of HAM/TSP patients and asymptomatic HTLV-1 carriers originating from the Kyushu area. The area of southern Kyushu in southwestern Japan is hyperendemic for HTLV-1 infection. The genetic background of the population in the southern Kyushu area is slightly different from that of the mainland Japanese population (19). We succeeded in establishing the largest DNA collection for HTLV-1 studies reported to date, consisting of 899 HAM/TSP patients and 753 asymptomatic HTLV-1 carriers. Using these DNA samples, we undertook a genome-wide association (GWA) study, a hypothesis-independent approach, to comprehensively identify genetic determinants for HAM/TSP.

## Results

### GWA Study for HAM/TSP in the Japanese Population.

**Fig. 1.**
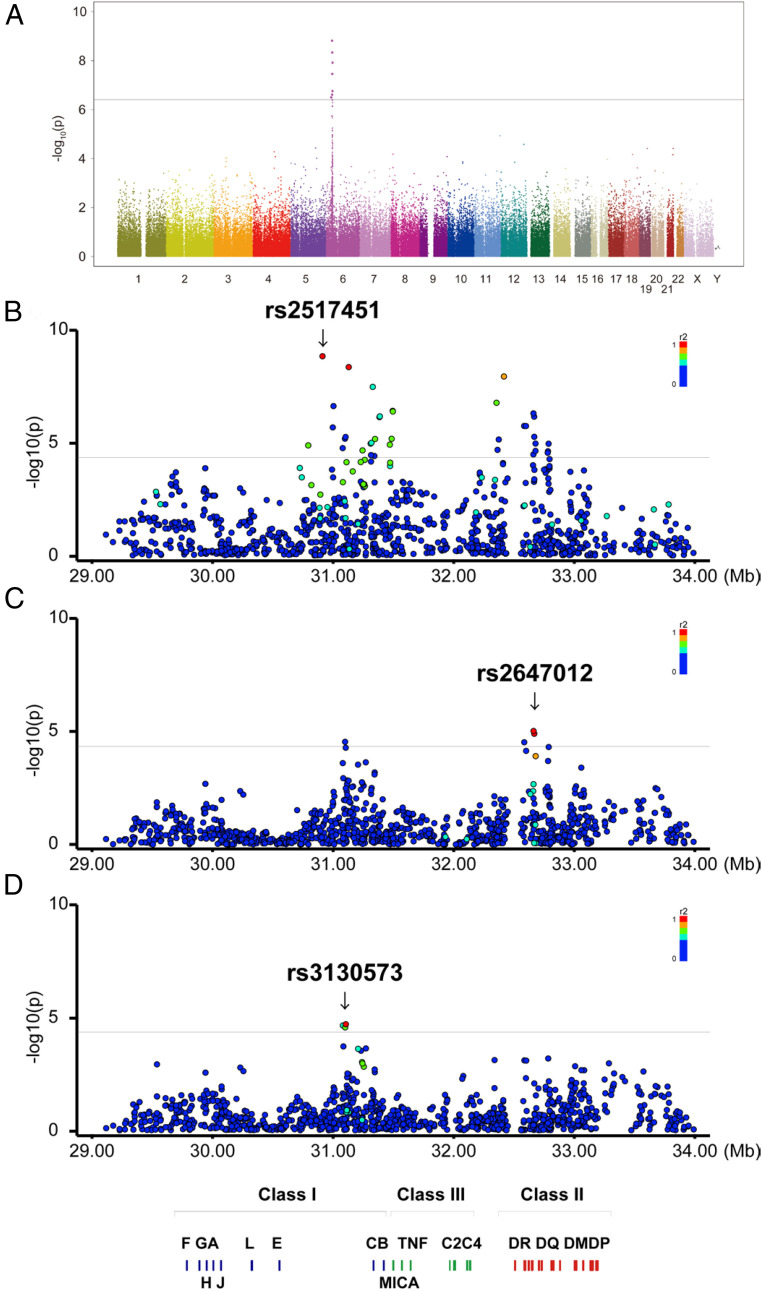
Manhattan plot and susceptible SNPs elucidated by the GWA studies. (*A*) The *P* values for genotyped SNPs from the GWA study for HAM/TSP patients and HTLV-1 carriers conditioned by 10 PCs are plotted along the chromosome in −log10 scale. The horizontal line indicates Bonferroni’s significance threshold (*P* = 3.95 × 10^−7^). (*B–D*) Regional Manhattan plots focusing on the HLA region conditioned by 10 PCs without SNP conditioning (*B*); 10 PCs and rs2517451 (*C*); and 10 PCs, rs2517451, and 2647012 (*D*). The color of each dot indicates LD (r2) from the top significant SNP in each study. Bonferroni’s significance threshold was set to the study-wide level: *P* = 4.58 × 10^−5^.

This GWA study was performed using DNA samples of 731 HAM/TSP patients and 846 asymptomatic HTLV-1 carriers for 126,394 single nucleotide polymorphisms (SNP) markers. A significant association peak was observed in the *HLA* locus on chromosome 6 (Fig. 1*A*). A Manhattan plot of the *HLA* locus revealed association signals in both *HLA* class I and class II loci (Fig. 1*B*). The strongest association signal was located in the vicinity of the *HLA-B* and *-C* genes (*P* = 1.54 × 10^−9^ for rs2517451), while the second peak was around the *HLA-DRA1* gene (*P* = 1.21 × 10^−8^ for rs28895103) (Table 1). SNP markers with *P* < 1.0 × 10^−5^ are listed in *SI Appendix*, Table S1.

**Table 1.** Genetic variations showing significant associations with HAM/TSP

SNP ID	Chr.	Position	A1/A2	Corresponding gene	Genotyping results*		OR (95% CI)^†^	*P* value‡
					HAM/TSP	Asymptomatic		
rs2517451	6	30914751	C/T	*DPCR1*	5/125/600	0/63/782	2.69	1.54 × 10^−9^
					(0.09)	(0.04)	(1.95–3.71)	
rs3130933	6	31132085	T/C	*POU5F1*	7/136/588	0/75/771	2.44	4.63 × 10^−9^
					(0.10)	(0.04)	(1.81–3.29)	
rs28895103	6	32419464	A/G	*HLA-DRA*	10/137/582	1/80/762	2.28	1.21 × 10^−8^
					(0.11)	(0.05)	(1.72–3.03)	
rs2523554	6	31331829	C/T	*DHFRP2*	25/206/500	6/167/673	1.83	3.48 × 10^−8^
					(0.18)	(0.11)	(1.48–2.27)	
rs12529049	6	32357715	T/C	*BTNL2*	13/167/551	6/108/732	1.92	1.76 × 10^−7^
					(0.13)	(0.07)	(1.50–2.46)	
rs2844670	6	31005726	G/A	*LOC729792*	67/335/329	36/318/491	1.56	2.46 × 10^−7^
					(0.32)	(0.23)	(1.32–1.84)	
rs13195509	6	26463660	A/G	*BTN2A1*	3/91/636	0/44/800	2.67	3.19 × 10^−7^
					(0.07)	(0.03)	(1.83–3.89)	

Chr., Chromosome.

* Genotype distribution (A1A1/A1A2/A2A2) is shown with frequency of A1 allele.

† OR and 95% CI are calculated for A1.

^‡^ Significant associations are defined as *P* < 3.95 × 10^−7^.

To further evaluate these associations in the *HLA* locus, we performed a forward stepwise multiple logistic regression analysis. The analysis conditioned on rs2517451 detected rs2647012 around the *HLA-DQB1* gene region as the next most significantly associated SNP (*P* = 9.49 × 10^−6^; Fig. 1*C*), and a subsequent analysis conditioned on rs2517451 and rs2647012 identified rs3130573 (*P* = 2.04 × 10^−5^; Fig. 1*D*). No additional SNP markers remained significant (*P* > 4.58 × 10^−5^) after conditioning on the above three markers.

### Genotyping of *HLA* Alleles and Association Analysis with HAM/TSP.

We next conducted association analyses of *HLA-A*, *-B*, *-C*, *-DRB1*, *-DQB1*, and *-DPB1* with HAM/TSP by genotyping these six *HLA* genes using an NGS-based targeted sequencing method. Frequencies of *HLA* alleles were compared between 651 HAM/TSP cases and 804 asymptomatic HTLV-1 carriers. Significant risk associations with HAM/TSP (*P* < 2.02 × 10^−4^) were observed for HLA-C*07:02 (*P* = 2.61 × 10^−5^), HLA-B*07:02 (*P* = 4.97 × 10^−10^), HLA-DRB1*01:01 (*P* = 1.15 × 10^−9^), and HLA-DQB1*05:01 (*P* = 2.30 × 10^−9^) (Table 2 and *SI Appendix*, Table S2). On the other hand, HLA-B*40:06 (*P* = 3.03 × 10^−5^), HLA-DRB1*15:01 (*P* = 1.06 × 10^−5^), and HLA-DQB1*06:02 (*P* = 1.78 × 10^−6^) showed a protective association with HAM/TSP. Haplotype analysis showed that susceptible alleles HLA-C*07:02, HLA-B*07:02, and HLA-DQB1*05:01 were on the haplotypes containing DRB1*01:01 (*SI Appendix*, Fig. S1). Similarly, HLA-DQB1*06:02 was on the haplotypes containing HLA-DRB1*15:01.

**Table 2.** List of *HLA* alleles showing significant association with HAM/TSP

Allele	HAM/TSP		Asymptomatic		F test	OR (95% CI)
	Count (*n* = 1,302)	Frequency	Count (*n* = 1,608)	Frequency		
C*07:02	198	0.152	161	0.100	2.61 × 10^−5^	1.61 (1.29–2.01)
B*07:02	137	0.105	72	0.045	4.97 × 10^−10^	2.51 (1.87–3.37)
B*40:06	40	0.031	103	0.064	3.03 × 10^−5^	0.46 (0.32–0.67)
DRB1*01:01	150	0.115	85	0.053	1.15 × 10^−9^	2.33 (1.77–3.08)
DRB1*15:01	56	0.043	134	0.083	1.06 × 10^−5^	0.49 (0.36–0.68)
DQB1*05:01	145	0.111	82	0.051	2.30 × 10^−9^	2.33 (1.76–3.09)
DQB1*06:02	43	0.033	118	0.073	1.78 × 10^−6^	0.43 (0.30–0.62)

Significant association was defined as *P* < 2.02 × 10^−4^ after Bonferroni correction.

**Fig. 2.**
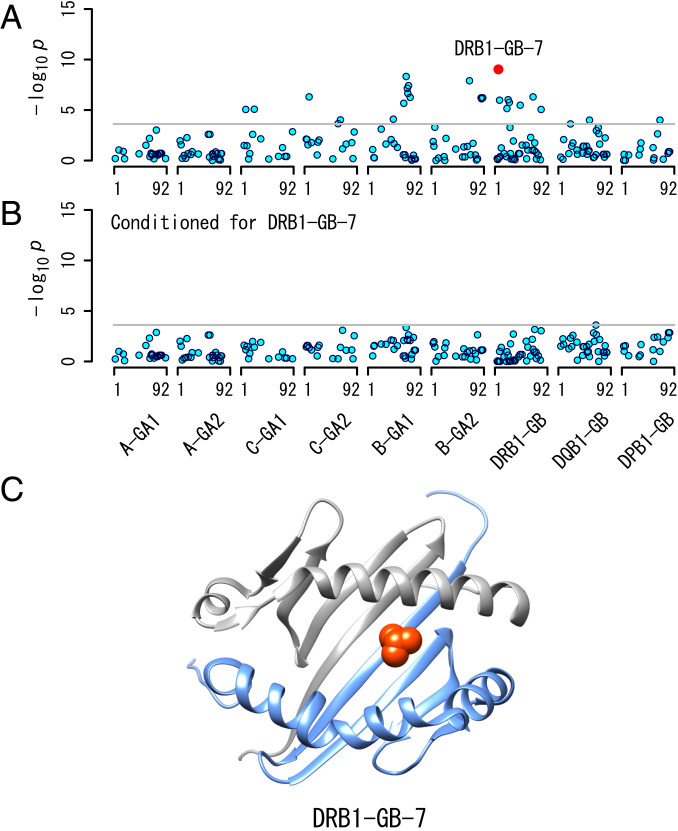
Results of association analysis of amino acid residues for HAM/TSP development across antigen presentation groove domains in the six classical HLA proteins. (*A*) Association analysis by logistic regression analysis of amino acid residues without any conditions. DRB1-GB-7 showed the most significant association with HAM/TSP. (*B*) Results of association analysis of amino acid residues with conditioning on DRB1-GB-7. There is no significant association after the conditioning. (*C*) The three-dimensional structure of HLA-DR protein. DRB1-GB-7, indicated by red spheres, is located in the beta sheet of the peptide-binding grooves.

We then performed forward stepwise logistic regression analysis for amino acid residues of the antigen presentation groove domains (G-DOMAINs). Amino acid position 7 of the G-BETA domain in *HLA-DRB1* (hereafter DRB1-GB-7) showed the strongest association (*P* = 9.52 × 10^−10^) (Fig. 2*A*). After conditioning on DRB1-GB-7, no other positions remained significant (*P* > 2.43 × 10^−4^; Fig. 2*B*). This amino acid position is in the β-sheet domain of the peptide-binding groove (Fig. 2*C*).

Additionally, we examined associations of amino acid residues at DRB1-GB-7 with HAM/TSP by multiple logistic regression analysis. We found that leucine at this position (DRB1-GB-7-Leu) was associated with the risk of HAM/TSP (*P* = 6.47 × 10^−7^, OR = 2.11, 95% CI = 1.57 to 2.83), whereas proline (DRB1-GB-7-Pro) worked protectively (*P* = 6.55 × 10^−5^, OR = 0.65, 95% CI = 0.53 to 0.8). Of note, the HLA-DRB1*01:01 susceptible allele carries DRB1-GB-7-Leu and the HLA-DRB1*15:01 protective allele encodes DRB1-GB-7-Pro (Table 3). HLA-DRB1*15:02 and HLA-DRB1*16:02 also carry DRB1-GB-7-Pro, although they did not show significant associations with HAM/TSP in this study.

**Table 3.** Associations between HAM/TSP and susceptible amino acid residues

Amino acid residue	Frequency		*P* value*	OR (95% CI)	*HLA* alleles^†^
	HAM/TSP (*n* = 1,302)	Asymptomatic (*n* = 1,608)			
DRB1-GB-7					
Leu	0.115	0.053	**6.47 × 10**^**−7**^	2.11 (1.57–2.83)	*01:01
Ser	0.352	0.340	—	1.00	*11:01, *08:02, *08:03, *12:01, *12:02, *13:02, *14:03, *14:05, *14:06, *14:54
Val	0.245	0.239	0.936	0.99 (0.82–1.20)	*04:01, *04:03, *04:05, *04:06, *04:10
Asp	0.130	0.132	0.656	0.95 (0.75–1.20)	*09:01
Pro	0.157	0.234	**6.55 × 10**^**−5**^	0.65 (0.53–0.80)	*15:01, *15:02, *16:02

* Significant association was defined as *P* ≤ 0.01 based on Bonferroni correction and shown in bold type.

† *HLA* alleles with frequency of greater than 0.01 either in case or control population were shown.

### Influence of *HLA* Alleles on HTLV-1 Proviral Load.

A previous study reported that HTLV-1 proviral load was significantly higher in HAM/TSP patients than in asymptomatic carriers (11). We compared the HTLV-1 proviral load between 424 HAM/TSP patients and 585 asymptomatic HTLV-1 carriers and found that indeed the proviral load was significantly higher in HAM/TSP patients than in asymptomatic HTLV-1 carriers (*P* = 4.7 × 10^−42^; *SI Appendix*, Fig. S2). We then investigated whether DRB1-GB-7 was associated with proviral load by using 353 HAM/TSP patients and 536 asymptomatic HTLV-1 carriers in whom *HLA-DRB1* alleles were successfully determined. We found the maximum proviral load (median = 5.91, SD = 8.24) of all diplotypes occurred in individuals who were homozygous for DRB1-GB-7-Leu (*SI Appendix*, Fig. S3 and Table S3). By contrast, those who were homozygous for DRB1-GB-7-Pro had the lowest median proviral load (median = 1.94, SD = 6.50). The observed maximum proviral loads found in those individuals who carry DRB1-GB-7-Leu most likely reflects the high number of HAM/TSP patients who always have high proviral loads and who are carrying the DRB1*01:01 allele. Therefore, we performed an association analysis between proviral load and amino acid residues on HLA proteins in the 536 asymptomatic HTLV-1 carriers. Leucine at amino acid position 70 of G-BETA domain in HLA-DRB1 (hereafter DRB1-GB-70-Leu), not DRB1-GB-7-Leu showed the strongest association with proviral load (*P* = 7.61 × 10^−6^; *SI Appendix*, Fig. S4). *HLA-DRB1* alleles carrying DRB1-GB-70-Leu included DRB1*08:02, DRB1*08:03, and DRB1*14:03 (*SI Appendix*, Table S4). There were no additional amino acid residues significantly associated with proviral load after conditioning for DRB1-GB-70-Leu (*P* < 1.22 × 10^−4^; *SI Appendix*, Fig. S4). Regression of proviral loads by DRB1-GB-70-Leu demonstrated that those who were homozygous for DRB1-GB-70-Leu had significantly higher proviral loads than those who were heterozygous (*P =* 3.88 × 10^−6^; *SI Appendix*, Table S4 and Fig. S5).

### Combined Effects of DRB1-GB-7 and Proviral Load on Risk of HAM/TSP.

An association analysis of HAM/TSP combining associated amino acid residues and proviral load showed that both DRB1-GB-7-Leu and DRB1-GB-7-Pro are associated with HAM/TSP development independent of the effect of proviral load (Table 4). Notably, those individuals who were homozygous for DRB1-GB-7-Leu had a surprisingly high odds ratio (OR) (9.57, 95% CI = 2.49 to 63.59) compared to those with combinations of other amino acid residues. Both homozygous (OR = 0.65, 95% CI = 0.35 to 1.16) and heterozygous (OR = 0.65, 95% CI = 0.46 to 0.91) individuals for DRB1-GB-7-Pro have the same “protective” ORs. Accordingly, we estimated the development rate of HAM/TSP for each type of amino acid residue at DRB1-GB-7 with changing proviral load. The development rate of HAM/TSP increases as the proviral load rises for all types of amino acid residues (*SI Appendix*, Fig. S6). In particular, the development rate of HAM/TSP in individuals homozygous for DRB1-GB-7-Leu at median proviral load (6.26%) (development rate = 3.55%, 95% CI = 0.76 to 16.54%, relative risk [RR] = 14.21) is 2.15 times that of asymptomatic carriers at median proviral load (1.44%) (development rate = 1.65%, 95% CI = 0.35 to 7.68%, RR = 6.61). This rate of increase is higher than the 2.09-fold rate of increase in individuals homozygous or heterozygous for DRB1-GB-7-Pro developing HAM/TSP (development rate = 0.32%, 95% CI = 0.24 to 0.41%, RR = 1.26) and asymptomatic carriers (development rate = 0.15%, 95% CI = 0.12 to 0.20%, RR = 0.60). In addition, it is higher than the increased rate of 2.10-fold of all data between HAM/TSP patients (development rate = 0.42%, 95% CI = 0.36 to 0.50%, RR = 1.69) and asymptomatic carriers (development rate = 0.20%, 95% CI = 0.17 to 0.24%, RR = 0.80).

**Table 4.** Associations of HAM/TSP with proviral load and susceptible amino acid residues

Variable	Coefficient	SE	*P* value (coefficient)	OR (95% CI)	*P* value (log- likelihood test)
DRB1-GB-7-Leu					
Intercept	−0.978	0.104	**3.39 × 10**^**−21**^	0.38 (0.31–0.46)	5.36 × 10^−39^
L	0.820	0.229	**3.35 × 10**^**−4**^	2.27 (1.45–3.57)	
LL	2.259	0.788	**4.13 × 10**^**−3**^	9.57 (2.49–63.59)	
PVL (log10)	1.200	0.121	**5.22 × 10**^**−23**^	3.32 (2.64–4.25)	
DRB1-GB-7-Pro					
Intercept	−0.674	0.107	**3.52 × 10**^**−10**^	0.51 (0.41–0.63)	2.39 × 10^−35^
P	−0.430	0.174	**0.013**	0.65 (0.46–0.91)	
PP	−0.437	0.302	0.148	0.65 (0.35–1.16)	
PVL (log10)	1.155	0.118	**1.61 × 10**^**−22**^	3.17 (2.54–4.04)	

Significant association was defined as *P* < 0.017 based on Bonferroni correction and shown in bold type.

## Discussion

We have identified multiple *HLA* alleles associated with HAM/TSP—HLA-B*07:02, HLA-C*07:02, HLA-DQB1*05:01, and HLA-DRB1*01:01—as risk alleles and HLA-B*40:06, HLA-DQB1*06:02, and HLA-DRB1*15:01 as alleles showing a protective effect. An analysis of amino acid residues in the G-DOMAIN of HLA proteins identified the amino acid residues at DRB1-GB-7 as the most significantly associated with HAM/TSP. DRB1-GB-7-Leu, which is carried by the HLA-DRB1*01:01 risk allele, showed the strongest association with HAM/TSP. Because HLA-C*07:02, HLA-B*07:02, DRB1*01:01, and HLA-DQB1*05:01 together constitute a haplotype, it is most likely that DRB1*01:01 is the susceptible allele to HAM/TSP, because it contains DRB1-GB-7-Leu. By contrast, DRB1-GB-7-Pro was protective against the onset of HAM/TSP and was shared by multiple *HLA* alleles, including HLA-DRB1*15:01 and other, less frequent *DRB1* alleles, such as DRB1*15:02, DRB1*15:06, DRB1*15:61, and DRB1*16:02 (Table 3 and *SI Appendix*, Table S2). Although HLA-DRB1*15:01 is in strong linkage disequilibrium (LD) with HLA-DQB1*06:02, which was also found to be protective, this LD cannot explain the protective effect of HLA-B*40:06. Indeed, HLA-B*40:06 protein has been reported to have limited recognition of anchor motifs and epitopes of HTLV-1 Tax peptide compared to other HLA class I proteins, which is essential for generating anti–HTLV-1 Tax CD8^+^ cytotoxic T lymphocytes (17). Hence, the protective effect of HLA-B*40:06 is likely independent of that of HLA-DRB1*15:01.

A nationwide epidemiologic study of HAM/TSP in Japan reported an average duration between the onset and clinical diagnosis of 7.6 y, due mainly to the poor awareness of the disease because of its rarity (20). It was also shown that 75.1% of patients had moderate to severe motor disability at a median of 9 years after onset, which corresponds to an Osame Motor Disability Score (OMDS) of ≥5 (20). On the other hand, a recent clinical trial reported that mogamulizumab (anti-CCR4) improved the OMDS in HAM/TSP patients (21), with the most pronounced improvements in participants who had enrolled at an early stage of the disease (disease duration, <10 y; OMDS <5). The registry of HLTV-1 carriers has been established in some countries, including Japan, to track carriers predisposed to HAM/TSP under close observation by specialized doctors for early diagnosis. However, even in Japan, with 1.2 million HTLV-1–infected people, establishing such a structure is still incomplete, and small numbers of neurologists and hematologists consult with only a limited number of HTLV-1 carriers. Moreover, most carriers are not even aware of their HTLV-1 infection and currently have no contact with medical institutions. This is mainly because the risk assessment method for HTLV-1 carriers has not been established to date. The follow-up would be inefficient if not focused on high-risk carriers. It is also difficult for the government to accept this for implementation as medical care. The results obtained in this study provide essential evidence to contribute to the establishment of risk assessment for HTLV-1 carriers, which is crucial for creating an appropriate follow-up system for carriers.

HTLV-1 proviral load has been used as a classical risk marker for HAM/TSP. Although proviral load varies among carriers, it becomes stable within a few years after initial infection and then remains relatively constant for each infected subject (22). In this study, our association analysis showed susceptible and protective amino acid residues on DRB1-GB-7 that have effects on HAM/TSP development and differ from the predictive association of proviral load. These amino acid residues would be effective biomarkers to predict the risk of HAM/TSP development even in the absence of a high proviral load. To demonstrate the availability of these biomarkers, we estimated a HAM/TSP development rate for each type of amino acid residue at DRB1-GB-7 with changing proviral load. There was a 23.6-fold difference in the development rate between individuals homozygous for DRB1-GB-7-Leu at the median proviral load leading to HAM/TSP and individuals homozygous or heterozygous for DRB1-GB-7-Pro at the median proviral load who are asymptomatic carriers. The difference in the rate of HAM/TSP development was >2.10-fold when estimated with all data. Therefore, the screening of risk groups for HAM/TSP development needs to be more precise when using DRB1-GB-7 and proviral load biomarkers in combination than when using them independently.

The lifetime development rate of HAM/TSP is different between Japan and the Caribbean area (0.25% vs. 1.9%) (10). Multiple factors influence the onset of HAM/TSP, including host and viral genetic factors, proviral load, and environment and lifestyle. The frequency of HLA-DRB1*01 alleles, including HLA-DRB1*01:01, HLA-DRB1*01:02, and, less frequently, HLA-DRB1*01:03, composing DRB1-GB-7 is higher in the Caribbean Indian (7.8%), Caribbean Black (7.0%), Caribbean Hispanic (9.0%), and Costa Rica Mestizo (9.5%) populations (23) than in the southern Kyushu population (5.3% in asymptomatic carriers in this study; *SI Appendix*, Table S5). However, this level of variance does not seem to fully account for the difference. There should be more genes involved in HAM/TPS; however, it is difficult to identify them comprehensively with a relatively small sample size. Future multiethnic genomic studies in the two populations recruiting larger numbers of samples and more comprehensive approaches, such as whole-genome sequencing, will help identify common and population-specific genetic determinants that better explain the difference.

Another critical factor in the onset of HAM/TSP to consider is the genotype of HTLV-1. A Japanese study demonstrated that among two HTLV-1 genotypes, tax subtype A (taxA) and tax subtype B (taxB), the former was predominant in patients with HAM/TSP (24). Another report showed a significantly lower frequency of taxA in the southern Kyushu population (9%) (25) compared with the Caribbean population, in which the preponderant HTLV-1 subtype is the cosmopolitan HTLV-I subtype A containing taxA (10). This may also partly explain the difference in the lifetime development rate of HAM/TSP, although an integrative analysis by adding tax genotype information is needed.

The allele frequency of HLA-DRB1*01:01 among other alleles carrying DRB1-GB-7 is equally high throughout other HTLV-1 endemic areas, such as the Caribbean region, Africa, and South America (23), making this biomarker potentially useful worldwide. In addition, we identified the amino acid residue DRB1-GB-70-Leu as positively associating with the amount of proviral load itself. However, its contribution to the overall proviral load is relatively small, thus precluding its use as a predictive marker for proviral load.

In this study, we could not provide a replicate group despite extensive recruitment throughout Japan, because of the low development rate (0.25%) of HAM/TSP. Moreover, retrospective recruitment does not allow consideration of any short-term changes in proviral load and disease status. Continuous recruitment of asymptomatic carriers of HTLV-1 in a prospective study could resolve this issue.

## Materials and Methods

### Study Subjects.

A total of 753 HAM/TSP patients and 899 asymptomatic HTLV-1 carriers of Japanese descent were enrolled in the study. The diagnosis of HAM/TSP was made according to the World Health Organization diagnostic criteria (26). In accordance with the Declaration of Helsinki, this study was reviewed and approved by the Ethics Committees of Kyoto University, St. Marianna University, Kyoto Prefectural University of Medicine, Kansai Medical University, Saga University, Kagoshima University, The University of Tokyo, Nagasaki University, Imamura General Hospital, and Kumamoto University. All patients were fully informed of the purpose and procedures of this study, and written consent was obtained from each subject.

### GWA Study.

A genome scan was conducted for 753 DNA HAM/TSP samples as cases and 899 samples of asymptomatic HTLV-1 carriers as controls. Of these, 436 cases and 523 controls were then genotyped using the Illumina Human 610-Quad BeadChip array, and the other 317 cases and 376 controls were genotyped with the Illumina HumanCoreExome BeadChip array in accordance with the manufacturer’s protocols. A total of 141,192 SNP markers, which were included in both arrays, were analyzed for associations.

The genome-scanned samples went through two rounds of quality control before association analysis. Initially, 4 cases and 6 controls with a call rate <0.95, 3 cases and 1 control as population outliers, and 15 cases and 46 controls showing kinships were excluded. After this step, 731 HAM/TSP samples and 846 controls remained for the analysis. Subsequently, 4,255 SNPs with a genotype call <0.99, 10,515 SNPs with a minor allele frequency <0.01, and 28 with deviation from Hardy–Weinberg equilibrium (*P* < 1.0 × 10^−6^) were excluded, resulting in a total of 126,394 SNPs for the analysis. A slight population stratification was observed (λ_GC_ = 1.03; *SI Appendix*, Fig. S7) after correcting for population stratification with the first 10 principal components (PCs).

### Determination of *HLA* Alleles.

We determined alleles of six major *HLA* genes—*HLA-A*, *-B*, -*C*, -*DPB1*, -*DQB1*, and -*DRB1*—by NGS sequencing technology in combination with long PCR-based specific amplification of these genes (*SI Appendix*, *Supplementary Note*) (27). Among the DNA samples used for the GWA study, 659 HAM/TSP and 821 HTLV-1 carrier samples that met the quality requirements for long PCR were chosen for the analysis. *HLA* alleles of these samples with four-digit resolution were determined with HLA-HD (28). Only the samples in which all the alleles were determined by the genotyping were included for analysis. The six *HLA* genes were successfully typed for 651 HAM/TSP patients and 804 asymptomatic controls. Haplotypes composing the six *HLA* genes were estimated using the PHASE method (29) and visualized using Disentangler (30). The haplotype estimation was independently executed for the HAM/TSP and asymptomatic HTLV-1 carrier sample sets.

### Association Analysis of HAM/TSP with *HLA* Alleles and Amino Acid Sequence.

Fisher’s exact test was used for the derivation of susceptible or protective *HLA* alleles in HAM/TSP patients and asymptomatic HTLV-1 carriers. The allele frequencies were summarized in four-digit resolution. A total of 247 different alleles were identified in the six *HLA* genes at four-digit resolution, and thus the Bonferroni-adjusted *P* value threshold was set to *P* = 2.02 × 10^−4^.

We also performed forward stepwise logistic regression analysis to assess the level of association between amino acid positions and residues in the six genes and infection outcome (31). For amino acid analysis, amino acid sequences corresponding to the *HLA* alleles were aligned per *HLA* locus with the use of the IMGT/HLA database (32). As there were 206 positions contributing to variation in the antigen presentation groove domains (G-ALPHA1 and G-ALPHA2 domains for class I molecules and G-BETA domain for class II molecules) (33), the Bonferroni-corrected significance threshold was set to *P* = 2.43 × 10^−4^. ORs for amino acid residues in the significantly associated amino acid positions were estimated with multiple logistic regression.

### Proviral Load Measurement and Association Analysis.

The proviral load was measured by real-time PCR for 424 HAM/TSP patients and 585 asymptomatic HTLV-1 carriers (34). In brief, the pX region of HTLV-1 provirus and the *RAG1* gene in 250 ng of genomic DNA were quantified, and proviral load was calculated by a relative quantification method using an ATL cell line, TL-Om1, as a reference. For statistical analyses, we used samples of 353 HAM/TSP patients and 536 asymptomatic HTLV-1 carriers whose *HLA* alleles and viral load were available. The samples were classified into six groups according to their genotypes for the seventh amino acid residue in the G-BETA domain of *HLA-DRB1*, and the association with proviral load was tested by the Wilcoxon rank-sum test between these groups. Associations of HAM/TSP development with susceptible amino acids and proviral load were calculated using a binomial logistic regression model. In this model, proviral loads considered below the detection limitation were set to 1.0 × 10^−4^%. Associations between proviral load and amino acid residues were calculated by a generalized linear regression model with quasi-Poisson distributions by amino acid residues with an additive model using asymptomatic HTLV-1 carriers only.

The development rate and RR of HAM/TSP for elucidated susceptible and protective amino acid residues were estimated using a binomial logistic regression model with changing proviral load value as a covariate. We set 0.25% as the baseline of development risk 1.0 to calculate RR.

## Supplementary Material

Supplementary File

## Data Availability

The whole set of association study results is available through the Human Genetic Variation Database (https://www.hgvd.genome.med.kyoto-u.ac.jp/repository; accession no. HGV0000015) (35). All other study data are included in the main text and *SI Appendix*.
