# Supplementary material for: Genome wide association study of HTLV-1–associated myelopathy/tropical spastic paraparesis in the Japanese population
Source: Proc Natl Acad Sci U S A. 2021 Mar 1;118(11):e2004199118. doi: 10.1073/pnas.2004199118 (PMC7980450; doi:10.1073/pnas.2004199118)
Supplement: Supplementary File [file pnas.2004199118.sapp.pdf]

Supplementary Information for

Genome wide association study of HTLV-1-associated myelopathy/tropical spastic paraparesis in the Japanese population

Authors: Marina Penova, Shuji Kawaguchi, Jun-ichirou Yasunaga, Takahisa Kawaguchi, Tomoo Sato, Meiko Takahashi, Masakazu Shimizu, Mineki Saito, Kunihiro Tsukasaki, Masanori Nakagawa, Norihiro Takenouchi, Hideo Hara, Eiji Matsuura, Satoshi Nozuma, Hiroshi Takashima, Shuji Izumo, Toshiki Watanabe, Kaoru Uchimaru, Masako Iwanaga, Atae Utsunomiya, Yasuharu Tabara, Richard Paul, Yoshihisa Yamano, Masao Matsuoka, Fumihiko Matsuda

Email: fumi@genome.med.kyoto-u.ac.jp

This PDF file includes:

- Figures S1 to S7
- Tables S1 to S5
- Supplementary text
- SI Reference

Table S1. List of the SNP markers showing *p*-values less than 1.0×10^{-5} in the GWA studies

SNP ID	Chr.	Position	Gene	Location	Nucleotide		Amino acid		Case				Control				*p*-value	OR (95% CI)
					Ref. (A1)	Var. (A2)	Ref.	Var.	A1/A1	A1/A2	A2/A2	A1 Freq.	A1A1	A1A2	A2A2	A1 Freq.		
rs2517451	6	30914751	*DPCR1*	intergenic	C	T			5	125	600	0.09	0	63	782	0.04	1.54×10^{-9}	2.69 (1.95-3.71)
rs3130933	6	31132085	*POU5F1*	3'UTR	T	C			7	136	588	0.10	0	75	771	0.04	4.63×10^{-9}	2.44 (1.81-3.29)
rs28895103	6	32419464	*HLA-DRA*	intergenic	G	A			582	137	10	0.89	762	80	1	0.95	1.21×10^{-8}	0.44 (0.33-0.58)
rs2523554	6	31331829	*DHFRP2*	intron	C	T			25	206	500	0.18	6	167	673	0.11	3.48×10^{-8}	1.83 (1.48-2.27)
rs12529049	6	32357715	*BTNL2*	intergenic	C	T			551	167	13	0.87	732	108	6	0.93	1.76×10^{-7}	0.52 (0.41-0.66)
rs2844670	6	31005726	*LOC729792*	intron	G	A			67	335	329	0.32	36	318	491	0.23	2.46×10^{-7}	1.56 (1.32-1.84)
rs13195509	6	26463660	*BTN2A1 V>M*	nonsynonymous	G	A	V	M	636	91	3	0.93	800	44	0	0.97	3.19×10^{-7}	0.37 (0.26-0.55)
rs3093983	6	31496925	*MCCD1 S>N*	nonsynonymous	G	A	S	N	12	178	541	0.14	5	125	716	0.08	3.98×10^{-7}	1.86 (1.47-2.37)
rs3093978	6	31498497	*MCCD1*	intron	C	A			12	178	541	0.14	5	125	715	0.08	4.26×10^{-7}	1.86 (1.46-2.37)
rs2647012	6	32664458	*HLA-DQB1*	intergenic	T	C			9	135	587	0.10	23	249	574	0.17	5.17×10^{-7}	0.56 (0.45-0.7)
rs2516448	6	31390410	*MICA*	intergenic	T	C			35	211	485	0.19	16	180	649	0.13	7.01×10^{-7}	1.65 (1.35-2.01)
rs2856717	6	32670308	*LOC100287443*	intergenic	A	G			9	135	587	0.10	23	247	575	0.17	7.24×10^{-7}	0.57 (0.45-0.71)
rs2596530	6	31387373	*MICA*	intergenic	G	A			35	211	485	0.19	16	180	648	0.13	7.56×10^{-7}	1.65 (1.35-2.01)
rs9461416	6	27825860	*HIST1H2BPS2*	intergenic	G	A			591	129	11	0.90	755	83	3	0.95	1.84×10^{-6}	0.51 (0.39-0.67)
rs9271366	6	32586854	*HLA-DQA1*	intergenic	G	A			8	175	548	0.13	26	288	532	0.20	1.87×10^{-6}	0.6 (0.49-0.74)
rs3104369	6	32602482	*HLA-DQA1*	intergenic	T	C			15	226	487	0.18	53	320	473	0.25	1.88×10^{-6}	0.64 (0.53-0.77)
rs10947121	6	30999997	*LOC729792 L>P*	nonsynonymous	T	C	L	P	79	333	319	0.34	47	328	470	0.25	2.16×10^{-6}	1.48 (1.26-1.74)
rs13192471	6	32671103	*LOC100287443*	intergenic	T	C			314	335	81	0.66	463	326	57	0.74	5.64×10^{-6}	0.69 (0.59-0.81)
rs3130573	6	31106268	*PSORS1C1*	intron	A	G			613	110	8	0.91	637	192	16	0.87	5.74×10^{-6}	1.73 (1.37-2.19)

rs3823418	6	31100942	*PSORS1C1*	intron	G	A		648	80	3	0.94	683	154	9	0.90	6.84×10^{-6}	1.9 (1.43-2.5)
rs3130637	6	31488145	*PPIAP9*	intron	A	G		13	187	530	0.15	7	138	701	0.09	6.92×10^{-6}	1.7 (1.35-2.13)
rs1521	6	31350704	*HLA-S*	intron	C	T		15	180	532	0.14	7	147	684	0.10	7.05×10^{-6}	1.69 (1.34-2.12)
rs3763313	6	32376471	*BTNL2*	intergenic	A	C		387	295	47	0.73	550	265	27	0.81	7.43×10^{-6}	0.67 (0.56-0.8)

OR: odds ratio, CI: confidence interval.

Table S2. Full list of HLA alleles included in the association tests (4-digit resolution).

Allele	HAM/TSP		Asymptomatic		*p* value	OR (95% CI)
	frequency	count	frequency	count	(F test)	
*A*02:01*	0.069	90	0.097	156	0.0073	0.69 (0.53-0.91)
*A*31:01*	0.096	125	0.073	117	0.0258	1.35 (1.04-1.76)
*A*02:06*	0.065	85	0.078	126	0.1957	0.82 (0.62-1.09)
*A*24:02*	0.444	578	0.424	682	0.2922	1.08 (0.94-1.26)
*A*26:03*	0.018	23	0.024	38	0.2988	0.74 (0.44-1.25)
*A*33:03*	0.045	59	0.054	87	0.3058	0.83 (0.59-1.16)
*A*26:02*	0.024	31	0.029	47	0.4195	0.81 (0.51-1.28)
*A*26:01*	0.085	111	0.077	124	0.4518	1.12 (0.85-1.46)
*A*11:01*	0.092	120	0.084	135	0.4684	1.11 (0.86-1.43)
*A*02:07*	0.029	38	0.032	51	0.7458	0.92 (0.60-1.41)
*C*07:02*	0.152	198	0.100	161	2.61×10^{-5}	1.61 (1.29-2.01)
*C*03:03*	0.102	133	0.138	222	0.0036	0.71 (0.57-0.89)
*C*08:01*	0.043	56	0.066	106	0.0073	0.64 (0.46-0.89)
*C*01:02*	0.241	314	0.208	335	0.0354	1.21 (1.01-1.44)
*C*14:02*	0.084	110	0.070	112	0.1405	1.23 (0.94-1.62)
*C*15:02*	0.019	25	0.025	40	0.3160	0.77 (0.46-1.27)
*C*14:03*	0.041	53	0.049	78	0.3241	0.83 (0.58-1.19)
*C*12:02*	0.118	154	0.130	209	0.3668	0.90 (0.72-1.12)
*C*08:03*	0.010	13	0.012	19	0.7222	0.84 (0.41-1.71)
*C*04:01*	0.029	38	0.032	51	0.7458	0.92 (0.60-1.41)
*C*03:04*	0.132	172	0.132	213	1.0000	1.00 (0.80-1.24)
*B*07:02*	0.105	137	0.045	72	4.97×10^{-10}	2.51 (1.87-3.37)
*B*40:06*	0.031	40	0.064	103	3.03×10^{-5}	0.46 (0.32-0.67)
*B*15:01*	0.035	46	0.062	100	0.0011	0.55 (0.39-0.79)
*B*15:18*	0.006	8	0.015	24	0.0306	0.41 (0.18-0.91)
*B*54:01*	0.132	172	0.109	175	0.0577	1.25 (1.00-1.56)
*B*39:01*	0.018	24	0.029	47	0.0696	0.62 (0.38-1.03)
*B*51:01*	0.098	128	0.081	131	0.1164	1.23 (0.95-1.59)
*B*40:01*	0.071	92	0.059	95	0.2239	1.21 (0.90-1.63)
*B*35:01*	0.068	89	0.080	129	0.2297	0.84 (0.64-1.11)
*B*59:01*	0.025	32	0.019	30	0.3024	1.33 (0.80-2.19)
*B*44:03*	0.041	53	0.049	78	0.3241	0.83 (0.58-1.19)
*B*52:01*	0.116	151	0.126	203	0.4247	0.91 (0.73-1.14)
*B*55:02*	0.034	44	0.029	46	0.4517	1.19 (0.78-1.81)
*B*15:11*	0.012	16	0.009	15	0.4713	1.32 (0.65-2.68)

*B*48:01*	0.017	22	0.019	31	0.6775	0.87 (0.50-1.52)
*B*40:02*	0.077	100	0.081	131	0.6792	0.94 (0.72-1.23)
*B*56:01*	0.011	14	0.009	15	0.7115	1.15 (0.56-2.40)
*B*46:01*	0.054	70	0.051	82	0.7384	1.06 (0.76-1.47)
*DRB1*01:01*	0.115	150	0.053	85	1.15×10^{-9}	2.33 (1.77-3.08)
*DRB1*15:01*	0.043	56	0.083	134	1.06×10^{-5}	0.49 (0.36-0.68)
*DRB1*15:02*	0.105	137	0.135	217	0.0165	0.75 (0.60-0.95)
*DRB1*14:54*	0.023	30	0.037	60	0.0309	0.61 (0.39-0.95)
*DRB1*04:06*	0.014	18	0.024	39	0.0444	0.56 (0.32-0.99)
*DRB1*04:01*	0.005	7	0.012	19	0.0756	0.45 (0.19-1.08)
*DRB1*08:03*	0.106	138	0.087	140	0.0871	1.24 (0.97-1.59)
*DRB1*14:05*	0.018	23	0.027	43	0.1054	0.65 (0.39-1.09)
*DRB1*14:06*	0.021	27	0.013	21	0.1098	1.60 (0.90-2.84)
*DRB1*08:02*	0.045	59	0.035	57	0.1831	1.29 (0.89-1.87)
*DRB1*14:03*	0.027	35	0.020	32	0.2165	1.36 (0.84-2.21)
*DRB1*04:05*	0.169	220	0.153	246	0.2429	1.13 (0.92-1.37)
*DRB1*12:02*	0.010	13	0.014	22	0.3968	0.73 (0.36-1.45)
*DRB1*04:10*	0.023	30	0.019	30	0.4329	1.24 (0.74-2.07)
*DRB1*12:01*	0.021	27	0.017	27	0.4903	1.24 (0.72-2.12)
*DRB1*13:02*	0.041	54	0.047	75	0.5270	0.88 (0.62-1.26)
*DRB1*16:02*	0.008	11	0.011	17	0.7032	0.80 (0.37-1.71)
*DRB1*11:01*	0.027	35	0.024	39	0.7227	1.11 (0.70-1.76)
*DRB1*09:01*	0.124	161	0.127	205	0.7788	0.97 (0.77-1.20)
*DRB1*04:03*	0.021	27	0.022	35	0.8977	0.95 (0.57-1.58)
*DQB1*05:01*	0.111	145	0.051	82	2.30×10^{-9}	2.33 (1.76-3.09)
*DQB1*06:02*	0.033	43	0.073	118	1.78×10^{-6}	0.43 (0.30-0.62)
*DQB1*05:03*	0.030	39	0.046	74	0.0265	0.64 (0.43-0.95)
*DQB1*05:02*	0.019	25	0.029	46	0.1163	0.66 (0.41-1.09)
*DQB1*03:01*	0.109	142	0.094	151	0.1932	1.18 (0.93-1.50)
*DQB1*04:01*	0.180	234	0.162	260	0.2145	1.14 (0.94-1.38)
*DQB1*04:02*	0.050	65	0.042	67	0.3244	1.21 (0.85-1.71)
*DQB1*06:04*	0.035	46	0.041	66	0.4399	0.86 (0.58-1.26)
*DQB1*03:02*	0.066	86	0.073	117	0.5104	0.90 (0.68-1.20)
*DQB1*06:01*	0.202	263	0.211	340	0.5500	0.94 (0.79-1.13)
*DQB1*03:03*	0.150	195	0.158	254	0.5704	0.94 (0.77-1.15)
*DPB1*02:01*	0.185	241	0.234	377	0.0012	0.74 (0.62-0.89)
*DPB1*04:02*	0.114	149	0.080	129	0.0019	1.48 (1.16-1.90)
*DPB1*03:01*	0.058	75	0.037	59	0.0097	1.60 (1.13-2.28)

*DPB1*19:01*	0.013	17	0.005	8	0.0249	2.65 (1.14-6.15)
*DPB1*13:01*	0.011	14	0.021	33	0.0390	0.52 (0.28-0.97)
*DPB1*09:01*	0.081	105	0.102	164	0.0534	0.77 (0.60-1.00)
*DPB1*14:01*	0.028	37	0.020	32	0.1425	1.44 (0.89-2.33)
*DPB1*05:01*	0.426	555	0.402	646	0.1852	1.11 (0.95-1.28)
*DPB1*04:01*	0.032	42	0.041	66	0.2369	0.78 (0.53-1.15)
*DPB1*135:01*	0.007	9	0.011	18	0.2500	0.61 (0.28-1.37)
*DPB1*02:02*	0.029	38	0.035	56	0.4015	0.83 (0.55-1.27)

HLA alleles with frequencies of either HAM/TSP or asymptomatic are equal or greater than 0.01.

OR: odds ratio, CI: confidence interval.

Fig. S1. Linkage disequilibrium plot between HLA alleles for HAM/TSP patients. Alleles are colored by the following definitions, light red: risk allele that has DRB1-GB-7-Leu, dark red: risk alleles that do not have DRB1-GB-7-Leu, light blue: protective allele that has DRB1-GB-7-Pro, dark blue: protective alleles that do not have DRB1-GB-7-Pro.

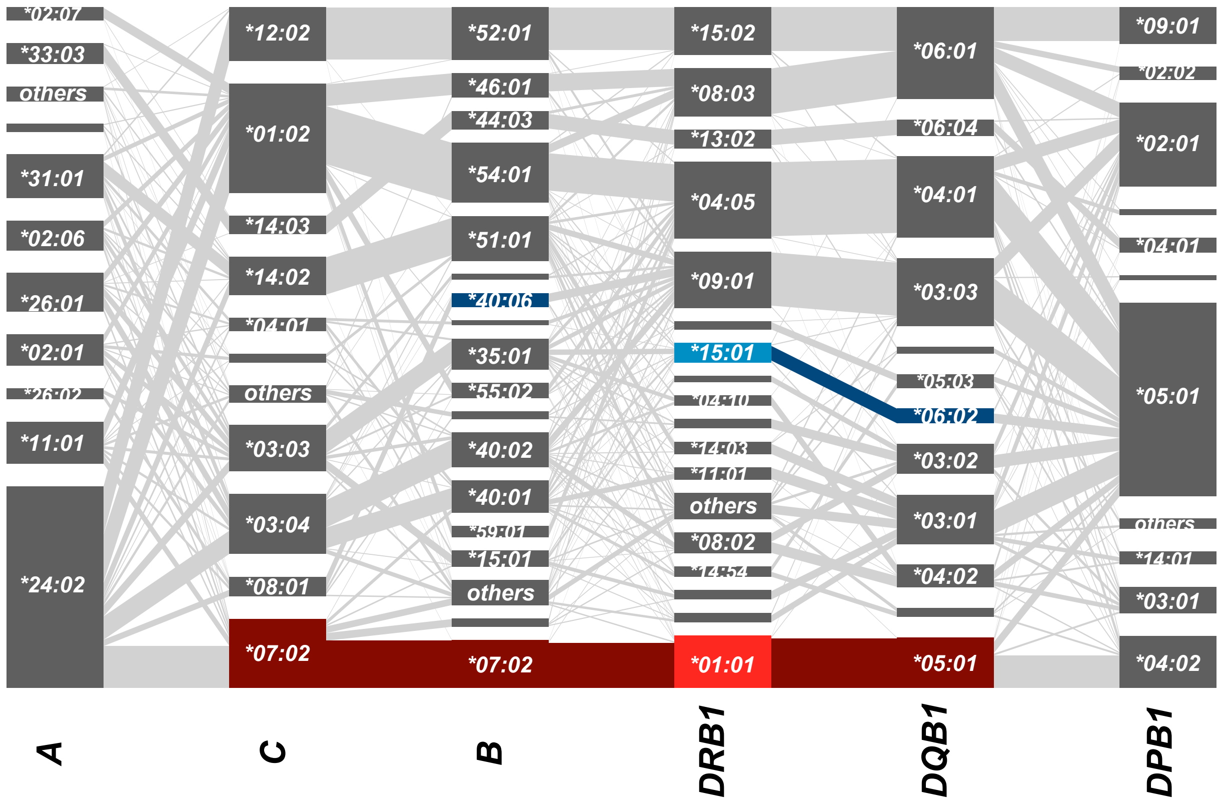

Fig. S2. Results of the two-sided Wilcoxon's rank sum test for proviral load between HAM/TSP patients and asymptomatic carriers.

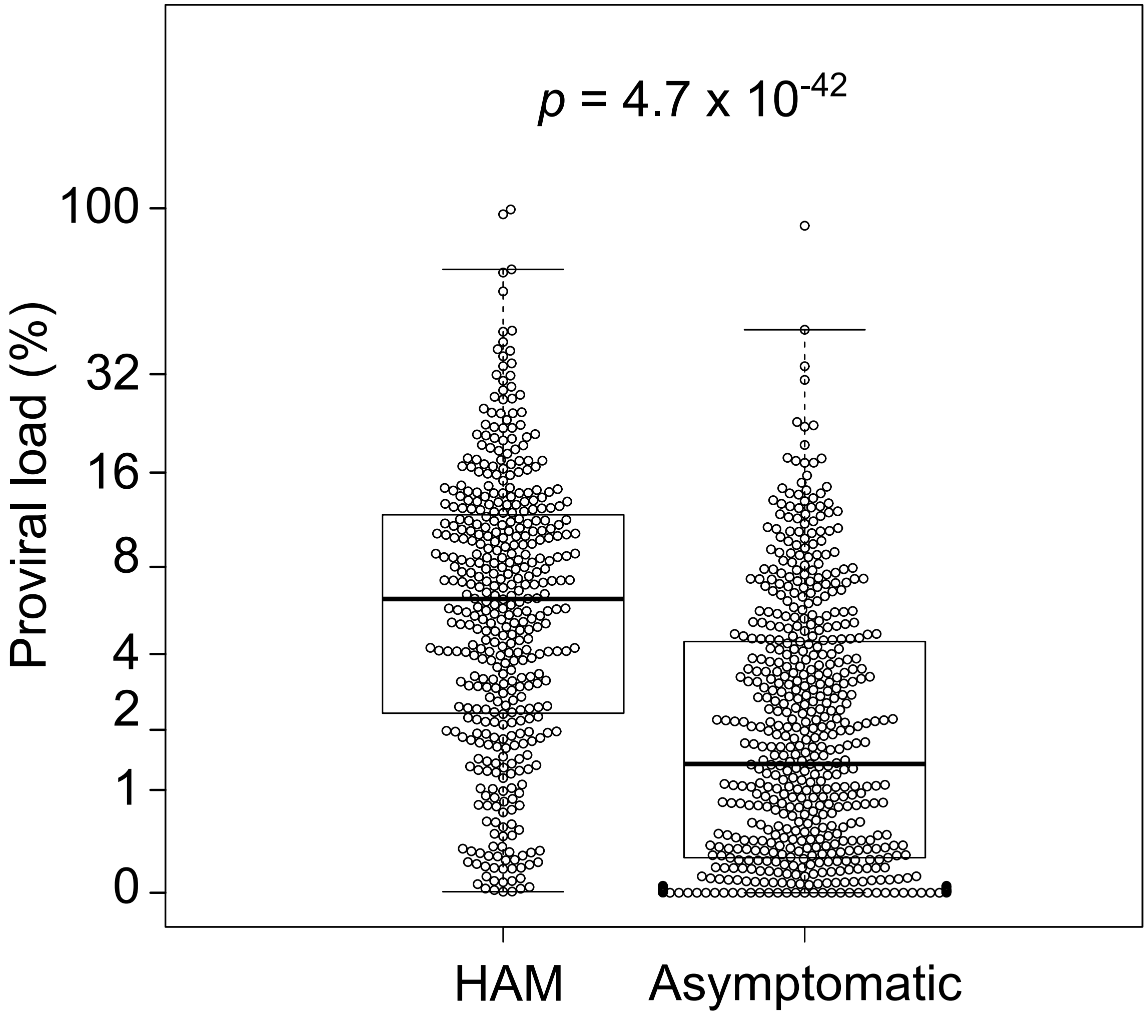

Fig. S3. Box plots of HTLV-1 proviral load for each diplotype of amino acid residues on DRB1-GB-7. Homozygotes for DRB1-GB-7-Leu and DRB1-GB-7-Pro had highest and lowest median of proviral loads, respectively.

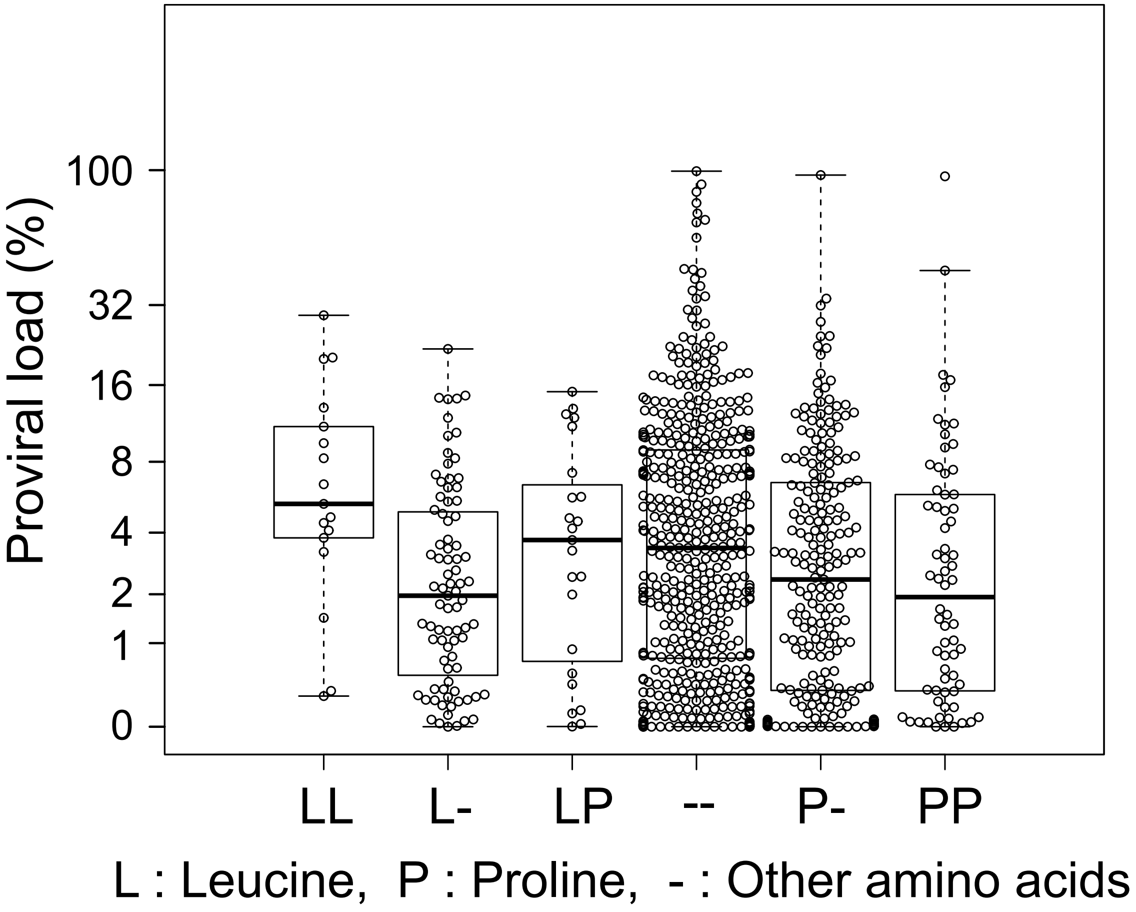

Table S3. Differences of proviral load between combination of amino acid residues at DRB1-GB-7.

Amino acid residues	Counts (frequency)		Median	*p*-value (Wilcoxon rank sum test)				
	HAM/TSP	Asymptomatic		L-	LP	--	P-	PP
LL	14 (0.040)	2 (0.004)	5.91	0.0028	0.1206	0.0720	0.0101	0.0059
L-	43 (0.122)	39 (0.073)	2.01	-	0.2764	0.0182	0.7115	0.7650
LP	12 (0.034)	11 (0.021)	3.70		-	0.8624	0.4211	0.2832
--	197 (0.558)	281 (0.524)	3.38			-	0.0095	0.0244
P-	67 (0.190)	157 (0.293)	2.42				-	0.5617
PP	20 (0.057)	46 (0.086)	1.94					-

L: leucine, P: proline, -: other amino acids.

Fig. S4. Results of association analysis between proviral load and amino acid residues across antigen presentation groove domains in the six classical HLA proteins. a) Associations between proviral load and amino acid residues without any conditions. DRB1-GB-70-Leu showed the most significant association with proviral load. b) Results of association analysis of amino acid residues with conditioning on DRB1-GB-70-Leu. There is no significant association after the conditioning.

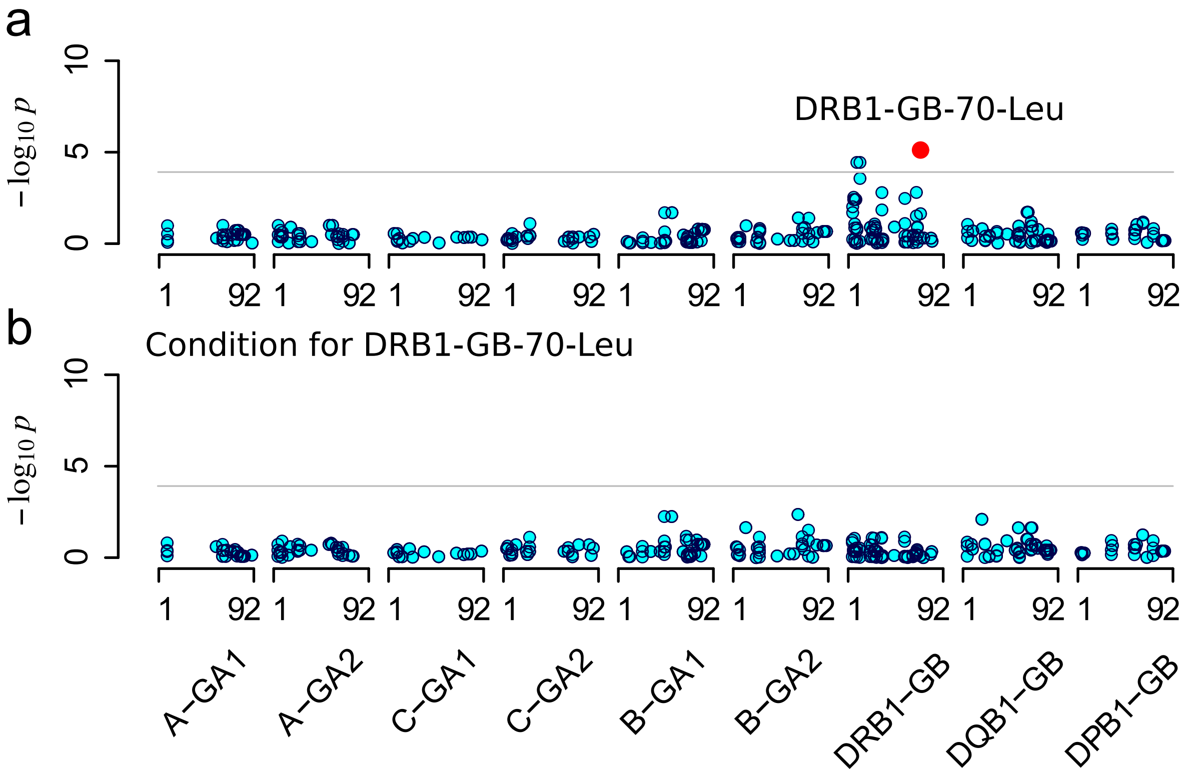

Table S4. Regression result of proviral load by susceptible HLA-DRB1-GB-70 amino acid residues.

DRB1-GB-70-Leu	Coefficient (95%CI)	Standard error	*p* value	*HLA-DRB1* alleles*
Intercept	1.10 (0.94-1.26)	0.080	**1.79×10^{-37}**	
L-	0.35 (0.06-0.64)	0.149	**0.0196**	**08:02*, **08:03*, **14:03*
LL	1.09 (0.63-1.55)	0.234	**3.88×10^{-6}**	

**HLA* alleles with frequency of greater than 0.01.

Fig. S5. Box plots of HTLV-1 proviral load for each diplotype of amino acid residues at DRB1-GB-70. Homozygotes for DRB1-GB-70-Leu had highest median of proviral loads.

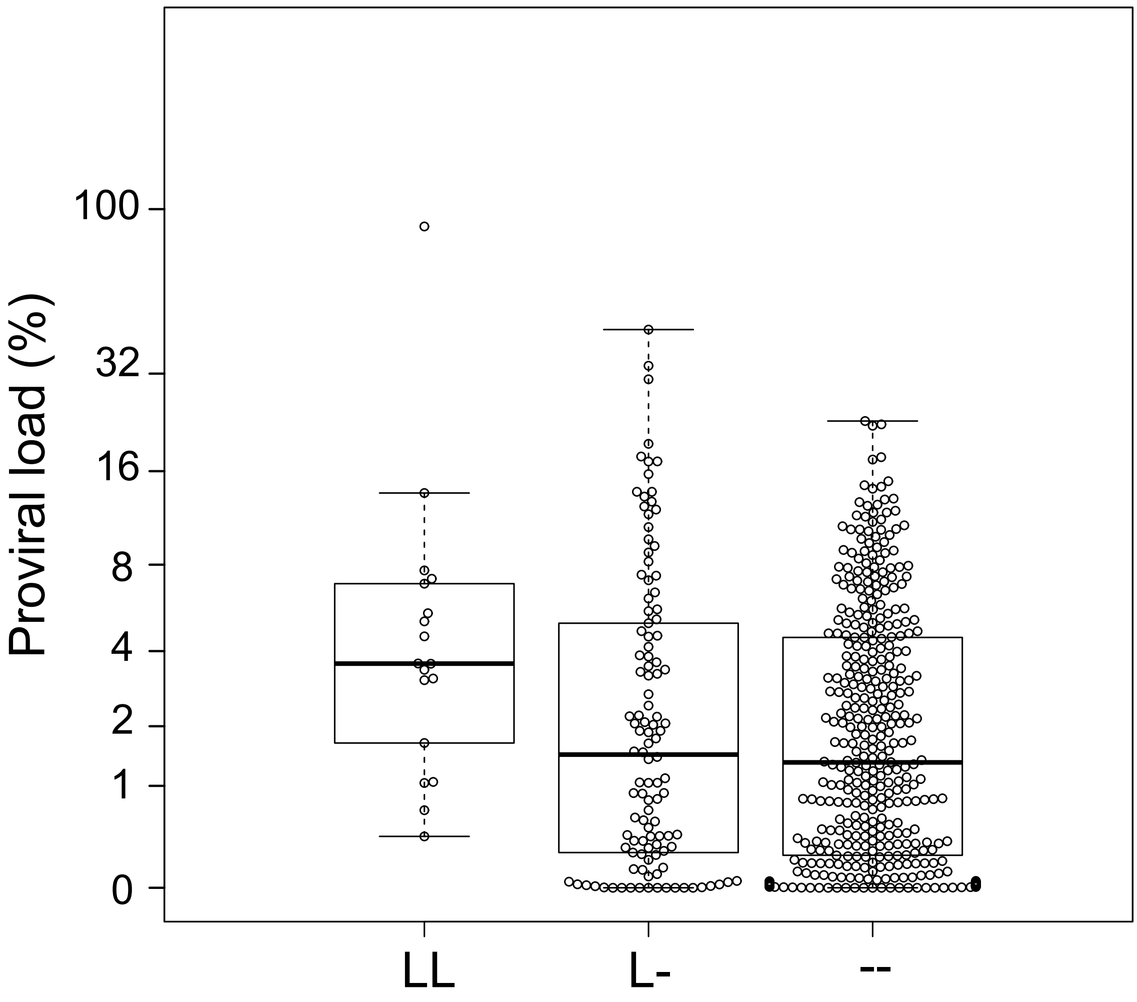

Fig. S6. Estimated curves of HAM/TSP development rate and relative risk for each type of amino acid residues at DRB1-GB-7 with changing proviral load. The basis of relative risk is set to 0.25%. Colored area covers 95% confidential intervals of development rates or relative risks.

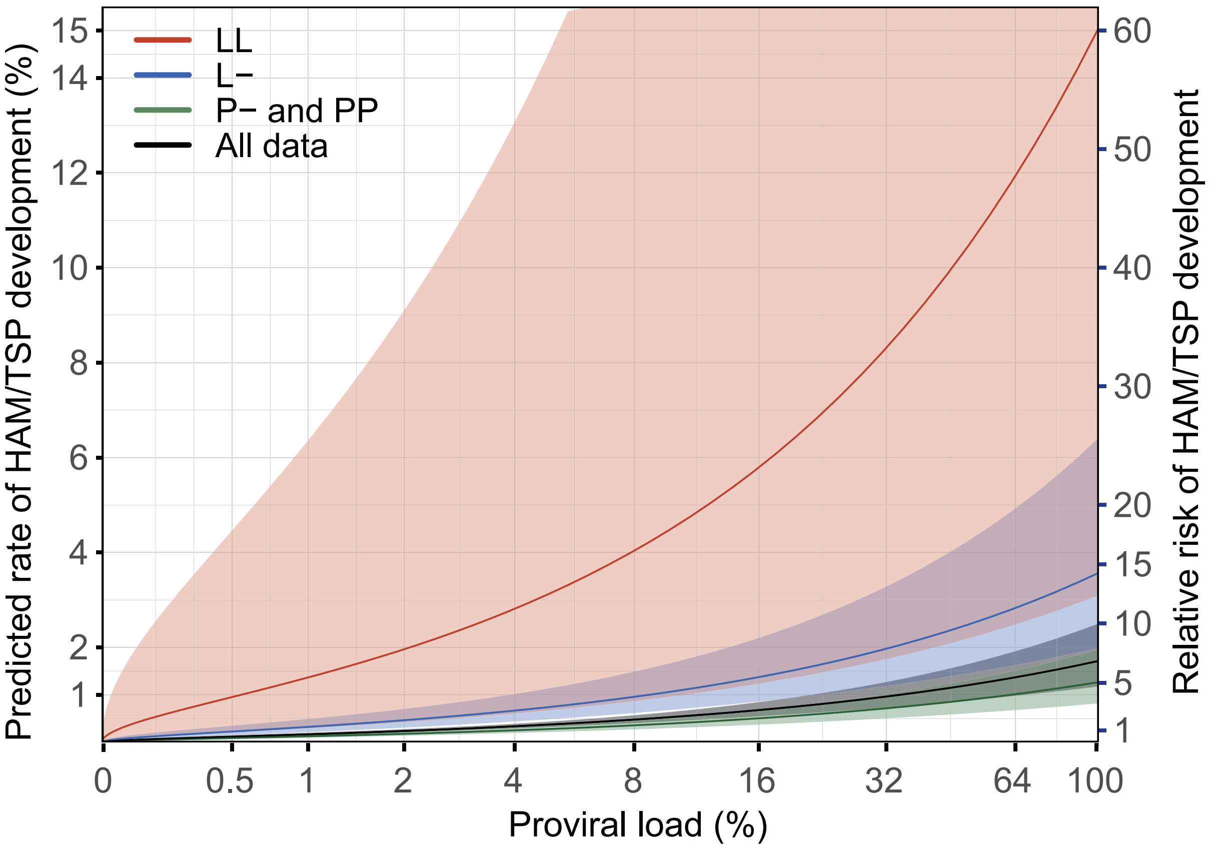

Table S5. Frequencies of DRB1*01 alleles in Japanese and Caribbean populations [23].

DRB1*01 allele	Southern Kyushu (n=804)	General Japanese * (n=18,604)	Caribbean Indian† (n=14,339)	Caribbean Black† (n=33,328)	Caribbean Hispanic† (n=115,374)	Costa Rica Mestizo # (n=331)
*DRB1*01:01*	0.0528	0.0582	0.0430	0.0243	0.0492	0.0560
*DRB1*01:02*	0.0000	0.0002	0.0330	0.0441	0.0366	0.0317
*DRB1*01:03*	0.0000	0.0000	0.0020	0.0017	0.0042	0.0073
Total	0.0528	0.0584	0.0780	0.0701	0.0900	0.0950

* Japan pop 16 population was used.

† USA NMDP population was used.

# Costa Rica Central Valley (n=221) and Guanacaste (n=110) Mestizo populations were merged.

Fig. S7. Q-Q plots of GWA study. Quantile-Quantile plots of the GWA study for HAM patients and HTLV1 carriers conditioned by 10PCs are plotted in anti-log scale (top) and log scale (bottom).

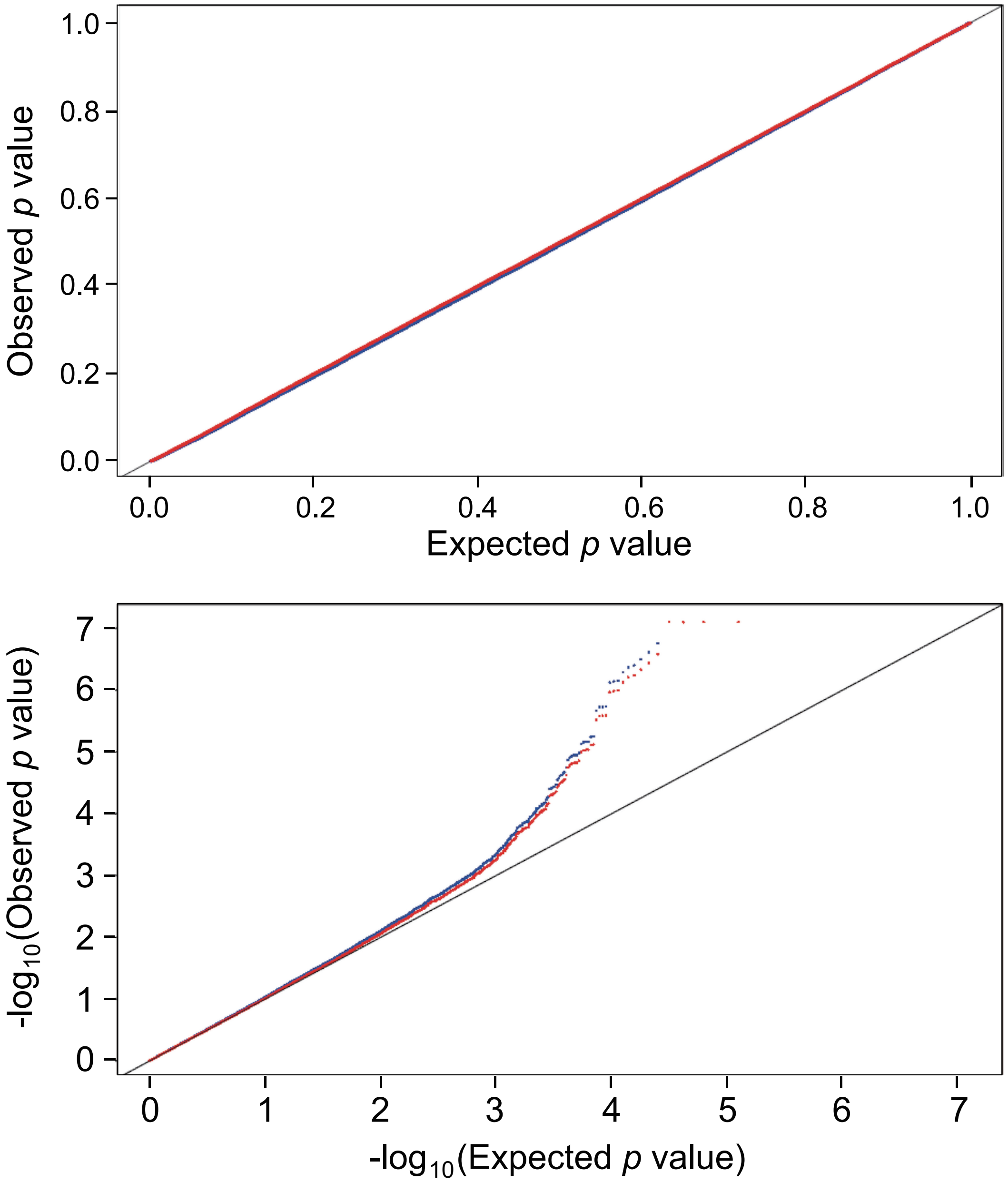

Supplementary Text. Protocol of PCR amplification and sequencing for *HLA* genes.

The six *HLA* genes were amplified by two different primer sets. Primer set A was developed by National Institute of Genetics[1] and one DRB1 reverse primer (GCATCCACAGAATCACATTTTCCAGTATT) was added. Set A was used for 414 HAM/TSP patients and 541 asymptomatic carriers. Primer set B is in-house development of Kyoto University and was used for 245 HAM/TSP patients and 280 asymptomatic carriers. Details of primer set B are shown in Table A. Each PCR was performed with 20 ng of genomic DNA, 0.5 units of PrimeSTAR® GXL DNA polymerase (TAKARA BIO INC., Japan), 1x PrimeSTAR® GXL buffer, 0.2 mM of each dNTP and specific amounts of each primer set reflecting the length of the amplified fragment (Table A) in a reaction volume of 20 μl. The PCR products for each sample were purified with Agencourt AMPure XP (Beckman Coulter, Inc.) and mixed in equimolar amounts. DNA libraries containing all six genes were prepared with the Nextera XT DNA sample preparation kit from Illumina using 0.5 ng of the amplified mix for each sample. The samples were double-indexed and size selection was performed with 0.5x volume Agencourt AMPure XP. Then they were pooled in sets for sequencing on the Illumina MiSeq platform. The Miseq flowcell of 2×300-bp paired-end reads resulted in 25-30 million total reads per run, corresponding to 14-17 gigabases of sequence data.

SI Reference

1 Hosomichi K, Jinam TA, Mitsunaga S, Nakaoka H, Inoue I. Phase-defined complete sequencing of the HLA genes by next-generation sequencing. *BMC Genomics* 2013; **14**: 355.

Table A. PCR primer set B for the six *HLA* genes.

HLA	Direction	Target exons	Reaction tube set	Sequence (5' to 3')
A	Forward	Exon 1-8	1	TTTCCAGAGAAGCCAATCAGTGTC
	Reverse		1	GTTTCTGCAAAGGCACCTGCATGTGTCTGT
C	Forward	Exon 1-8	1	GTCGGGTCCTTCTTCCTGAATACTCA
	Reverse		1	CGTTCCTGTTAGCATAATGTGAGGAGGTGG
B	Forward	Exon 1-7	2	GTCGGGTCCTTCTTCCAGGATACTCG
	Reverse		2	CAACTTCTTACTTCCCTACTGAAAATAAGA
DRB1	Forward	Exon 1-2	1	CCCTCCATCTCCTTTACTCC
	Reverse		1	CTGTTGTGGGAGGGGAGGCA
	Forward	Exon 2-6	2	AGGAGGACCTGTGAACCAGAG
	Reverse		2	ACAAACACATTACATTATTAAATGTTTCTCAAA
DQB1	Forward	Exon 1-7	2	GTCCTTCAGCTCCAGTGCTGATTGGTTC
	Reverse		2	CCACCAGGTCATCTCCTTTCATC
DPB1	Forward	Exon 1-2	1	CTTATCTGACTGGTTAAAATGAGTATCACT
	Reverse		1	GAGATGGATTTTACTTGTCTTGGGTTC
	Forward	Exon 2-5	2	CAGAGTCTTTCTTATACCAAAGTTGAAGAA
	Reverse		2	TCTTCAAATCGAGATCATTATGAAATCCTC
